# MDMA-assisted therapy as a treatment for major depressive disorder: proof of principle study

**DOI:** 10.1192/bjp.2025.10320

**Published:** 2025-11

**Authors:** Tor-Morten Kvam, Ivar W. Goksøyr, Justyna Rog, Inger-Tove Jentoft van de Vooren, Lowan Han Stewart, Ingrid Autran, Mark Berthold-Losleben, Lynn Mørch-Johnsen, René Holst, Ingmar Clausen, Ole A. Andreassen

**Affiliations:** Institute of Clinical Medicine, University of Oslo, Norway; Nordre Østfold DPS, Østfold Hospital Trust, Grålum, Norway; Oslo University Hospital, Oslo, Norway; Department of Mental Health, Norwegian University of Science and Technology, Trondheim, Norway; Department of Psychiatry, Østfold Hospital Trust, Grålum, Norway; Department of Clinical Research, Østfold Hospital Trust, Grålum, Norway

**Keywords:** Major depressive disorder, clinical drug studies, medication-assisted treatment, psychotherapy

## Abstract

**Background:**

3,4-methylenedioxymethamphetamine (MDMA)-assisted therapy (MDMA-AT) has shown promising safety and efficacy in phase 3 studies of post-traumatic stress disorder, but has not been investigated for a primary diagnosis of major depressive disorder (MDD).

**Aim:**

We aimed to explore the proof of principle and safety as a first study with MDMA-AT for MDD, and to provide preliminary efficacy data.

**Method:**

Twelve participants (7 women, 5 men) with moderate to severe MDD received MDMA in 2 open-label sessions 1 month apart, along with psychotherapy before, during and after the MDMA sessions, between January 2023 and May 2024. The primary outcome measure was mean change in Montgomery–Asberg Depression Rating Scale (MADRS), and the secondary outcome measure was mean change in functional impairment as measured with the Sheehan Disability Scale (SDS), both from baseline to 8 weeks following the second MDMA session. We used descriptive statistics and the two-tailed Wilcoxon signed-rank test to compare baseline and outcome scores. Repeated measures were analysed by a mixed-effects model.

**Results:**

Baseline MADRS was 29.6 (s.d. 4.9). Feasibility was demonstrated with sufficient recruitment and retention. MADRS scores were significantly reduced post treatment compared with baseline (mean difference –19.3, s.e. 2.4, CI –14.8 to –23.8, *P* < 0.001). SDS scores significantly decreased from baseline (mean difference –11.7, s.e. 2.2, CI –7.5 to –15.9, *P* = 0.001). There were no adverse events of special interest, and no unexpected or serious adverse events.

**Conclusion:**

The study met the primary objectives of safety and feasibility, and provided indications of efficacy for MDMA-AT for MDD. Further studies with a randomised design are required to confirm these findings.

**Trial registration:**

EudraCT no. 2021-000805-26.

Major depressive disorder (MDD) is a common disorder and a leading cause of disability.^1^ The available treatments have limited effectiveness and tolerability, and the relapse rate is high.^2–4^ 3,4-methylenedioxymethamphetamine (MDMA) is a stimulant with monoamine-releasing and reuptake inhibitor properties,^5–7^ and appears to induce neuroplasticity.^8^ While MDMA is a Schedule I drug with moderate abuse potential, the American Food and Drug Administration (FDA) has stated that MDMA has a satisfactory safety profile for testing in clinical trials.^9^ MDMA is hypothesised to act as a catalyst of the psychotherapeutic process, with subjective effects such as reduced anxiety, increased trust and insight^10^ and enhanced self-compassion, emotion regulation and other self-capacities.^11^ MDMA-assisted therapy (MDMA-AT) is a novel approach to the treatment of mental disorders. In this experimental treatment model, MDMA was administered with therapy in 2 or 3 monthly 8-hour sessions, in combination with preparatory and integrative non-drug therapy sessions.^12^ Preliminary results are promising for several indications,^13–15^ but post-traumatic stress disorder (PTSD) has been the primary focus in clinical trials. Two randomised, placebo-controlled, phase 3 trials have demonstrated the safety and efficacy of MDMA-AT for PTSD.^16,17^ However, a new drug application to the FDA was rejected and an additional phase 3 trial has been requested. In one of the phase 3 trials for PTSD, the MDMA group showed a significant reduction in depressive symptoms compared with placebo.^17^ Many MDD individuals have experienced traumatic events, and childhood trauma is associated with depression in adults.^18^ This overlap suggests that the effects observed in PTSD may apply to subjects with MDD. However, MDMA-AT therapy has not been studied as a treatment for individuals with a primary diagnosis of MDD. The primary objectives of the current study were to explore the feasibility and safety of MDMA-AT for MDD, and to gain a preliminary impression of treatment efficacy.

## Method

### Design

Because this is the first study to investigate MDMA-AT for MDD, an open-label proof of concept approach is justified. See the study protocol article for details.^19^

### Participants

Participants were recruited through self-referrals and referrals from general practitioners, psychiatrists or psychotherapists.

The inclusion criteria were (a) a diagnosis of single or recurrent episodes of MDD without psychotic features, with a duration >12 weeks and <2 years at time of enrollment, as defined in DSM-5; (b) at least moderate depression at time of enrollment (Montgomery–Asberg Depression Rating Scale (MADRS) ≥20); and (c) age ≥18 years.^19^

Exclusion criteria included psychotic disorders, mania, personality disorders, eating disorders with purging, substance use disorders, serious suicide risk, pregnancy, recent electroconvulsive therapy or ketamine, and medical conditions posing risks from increased blood pressure or heart rate;^19^ see also Supplementary material (available at https://doi.org/10.1192/bjp.2025.10320).

Participants were asked about their gender assigned at birth and their gender identity as separate questions.

### Procedures

An overview of the study visits is provided in Supplementary Fig. 1. Pre-study assessments are described in detail in the Supplementary material. If deemed initially eligible, participants signed the informed consent form after the procedures had been fully explained. Written informed consent was obtained from all patients.

Preparatory period and enrollment confirmation: during the preparatory phase, three 90-min sessions were conducted, along with a baseline visit. If MADRS score was 20 or higher at the baseline visit (visit 3 (V3)), enrollment was confirmed and further study visits were scheduled. Psychotropic medications were tapered following enrollment (see Supplementary text for details).

Treatment period: this consisted of two MDMA sessions approximately 4 weeks apart, each followed by 4 follow-up telephone calls the following week, and three 90-min integration sessions over 4 weeks. At the first MDMA dosing session, participants received an initial dose of 80 mg MDMA; at the second session, all participants accepted the offer of increasing the initial dose to 120 mg. At each dosing session a supplemental half of the initial dose was offered 90–120 min later to prolong the psychoactive effects of MDMA, thereby allowing for sustained psychotherapeutic processing, unless tolerability issues emerged following the initial dose or the participant declined. All participants, except for one, received the supplemental dose in both of the dosing sessions. See Supplementary Table 1 for an overview of the flexible doses offered during MDMA sessions 1 and 2. Approximately 8 h after the initial dose the participant’s condition was assessed by the study physician, and the session was ended if all medical and psychiatric parameters were acceptable. All participants spent the night at the study site. with optional support from a night attendant.

Psychotherapists: all therapists in the study were licensed health professionals: three psychologists and one psychiatrist. Three of the therapists were trained prior to a sponsored trial investigating MDMA-AT for PTSD.^20^ See Supplementary material for details regarding MDMA-AT and previous therapist training.

Raters: because this was an open-label trial, raters for the primary outcome measure were not fully blinded although they remained largely unaware of whether the assessment was pre or post treatment. The raters were relatively independent, being affiliated with the hospital but not with the study team itself.

Protocol deviations were logged and reviewed by the monitor.

### Outcome measures

Measures of feasibility: we collected recruitment and retention rates as indicators of feasibility. See the study protocol article for details regarding screening failures, early terminations, drop-outs and lost to follow-up.^19^

Safety outcome measures: adverse events were recorded by the study therapists in close collaboration with the sub-investigator, a psychiatrist. We tracked the outcome of adverse events, any treatments provided and any hospitalisation or discontinuation of MDMA-AT due to adverse events. We also monitored the incidence of treatment-emergent adverse events (TEAEs) and the use of psychotropic concomitant medications during the MDMA dosing sessions, and for the following 2 days, as well as throughout the entire treatment period. Suicidal ideation, the intensity of suicidal ideation and suicidal behaviour were measured with the Columbia-Suicide Severity Rating Scale (C-SSRS). Any deterioration in C-SSRS score from baseline was reported as an adverse event. Adverse events of special interest (AESIs) were related to cardiac function, serious suicide ideation or suicidal behaviour and potential for abuse, as defined in the protocol;’^19^ see also the Supplementary material. Furthermore, we evaluated mean changes in blood pressure, heart rate and body temperature before both the initial and supplemental doses, as well at the end of each MDMA dosing session.^19^

The primary outcome measure was change in MDD symptom severity, as measured by mean change in MADRS scores from baseline to outcome visit ∼8 weeks after the second MDMA session (∼12 weeks post baseline). The secondary outcome measure was change in functional impairment, as measured by mean change in Sheehan Disability Scale (SDS) score, from baseline to outcome visit. Exploratory outcome measures included changes in depression (Beck’s Depression Inventory II, BDI-II), insomnia (Bergen Insomnia Scale, BIS), post-traumatic stress symptoms (the PTSD Checklist for DSM-5, PCL-5), anxiety (Generalised Anxiety Disorder 7, GAD-7), the Alcohol Use Disorders Identification Test (AUDIT) and the Drug Use Disorder Identification Test (DUDIT). These exploratory outcome measures were collected at baseline visit (visit 4), except for AUDIT and DUDIT, which were collected during screening and the study termination visit (visit 14, ∼12 weeks post baseline). Responders were defined as those having a reduction of ≥50% in MADRS score, and remitters as those with a MADRS score ≤12 post treatment.

### Ethics and approvals

The authors assert that all procedures contributing to this work comply with the ethical standards of the relevant national and institutional committees on human experimentation, and with the Helsinki Declaration of 1975 as revised in 2013. All procedures involving patients were approved by the Norwegian Medicines Agency (reference no. 21/18379-18) and the Regional Committees for Medical and Health Research Ethics (application no. 145565).

### Statistics

Along with descriptive statistics, we used the non-parametric, two-tailed Wilcoxon signed-rank test to compare baseline and outcome scores, assuming non-normality. Additionally, we performed 4000 bootstrap resamples of mean change to estimate 95% CI. For repeated measures of BDI we used a mixed-effects model.

## Results

### Feasibility

**Fig. 1:**
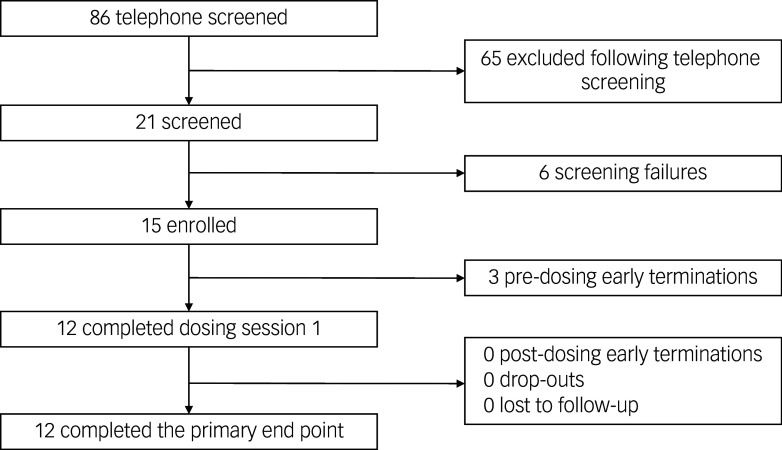
Recruitment and retention Consort flow diagram.

The study was open for recruitment between January 2023 and May 2024; recruitment to, and retention in, the study are shown in Fig. 1. The study flow diagram shows the key numbers for recruitment to the study, and the extent to which participants remained in the study as an indication of the tolerability of the treatment. There were no post-dosing early terminations, drop-outs or lost to follow-up. Eleven participants entered the study through self-referrals while one was referred by a study therapist. Although we received referrals from other healthcare professionals, none of those participants were considered eligible.

### Demographics and baseline characteristics

Demographics and baseline characteristics are shown in Table 1. There were 7 women in the sample, and mean age was 44.1 years (s.d. 9.2). All but one of the participants were Caucasian, and two were of non-Norwegian origin. One participant had a single episode of MDD while the remainder had recurrent MDD. The mean time since the first MDD episode was 16.1 years (s.d. 10.4). Baseline MADRS was 29.6 (s.d. 4.9). Ten participants had moderate severity of depression (defined as MADRS 20–34) while 2 had severe depression (defined as MADRS ≥35). All participants had previous psychotherapeutic experience, with a mean number of 5.5 therapies (s.d. 3.3) and 223.1 therapy sessions (s.d. 333.9), but the distribution was highly right-skewed (median 88.5, skewness 2.5). Visual inspection revealed no systematic association between baseline demographic characteristics and change in MADRS scores.

**Table 1:** Demographics and clinical characteristics of participants at baseline

Characteristics	Mean (s.d.)
Age (years)	44.1 (9.2)
Gender assigned at birth, *n* (%)	
Men	5 (41.7)
Women	7 (58.3)
Gender identity, *n* (%)	
Men	5 (41.7)
Women	7 (58.3)
Ethnicity, *n* (%)	
Non-Norwegian	2 (16.7)
Norwegian	10 (83.3)
Race, *n* (%)	
Asian	1 (8.3)
White	11 (91.7)
Body mass index (kg/m^2^)	26.9 (4.3)
Annual income (NOK)	507 500 (218 137.8)
Education (years),	18.6 (4.0)
Social security payments past 3 months	
*n* (%)	7 (58.3)
Payments (NOK) for those who received	75 457.1 (58 473.9)
Sick leave past 3 months	
*n* (%)	7 (58.3)
Days off work for those who were on sick leave	57.9 (29.8)
Primary diagnosis (ICD-10)	
Recurrent MDD (f33), *n* (%)	11 (91.7)
Single episode (f32), *n* (%)	1 (8.3)
Duration of MDD (years)	16.1 (10.4)
Psychiatric comorbidity	1.0 (1.2)
Psychiatric comorbidity, *n* (%)	
PTSD	3 (25.0)
ADHD	3 (25.0)
Social anxiety	1 (8.3)
Generalised anxiety disorder	1 (8.3)
Panic disorder with agoraphobia	1 (8.3)
Insomnia	1 (8.3)
Autism spectrum disorder	1 (8.3)
Previous use of antidepressants	
*n* (%)	9 (75.0)
*n* series, lifetime	2.8 (1.0)
≥2 previous antidepressant series (%)	8 (66.7)
Duration of treatments (months)	65.1 (92.3)
*n* series, current episode	0.7 (0.9)
Tapering of ongoing psychotropic medication, *n* (%)^a^	8 (66.7)
SSRIs	2 (16.7)
Other antidepressants^b^	4 (33.3)
Quetiapine	2 (16.7)
Other	1 (8.3)
Previous psychological treatments	
*n* series	5.5 (3.3)
*n* sessions (h)	223.1 (333.9)
Baseline MADRS score	29.6 (4.9)
Depression severity	
Moderate (MADRS 20–34), *n* (%)	10 (83.3)
Severe (MADRS ≥35), *n* (%)	2 (16.7)
Baseline SDS score	
Total score	21.3 (6.1)
Days lost at school or work	2.5 (2.2)
Underproductive days	6.1 (1.8)
Baseline C-SSRS score,	
Suicidal ideation, lifetime	3.4 (1.4)
Ideation intensity, lifetime	13.4 (4.0)
Suicidal ideation, past 6 months	1.7 (1.3)
Ideation intensity, past 6 months	7.2 (5.9)
Previous use of MDMA, *n* (%)	1.0 (8.3)

MDD, major depressive disorder; PTSD, post-traumatic stress disorder; ADHD, attention-deficit hyperactivity disorder; SSRIs, selective serotonin reuptake inhibitors; MADRS, Montgomery–Asberg Depression Rating Scale; C-SSRS, Columbia-Suicide Severity Rating Scale; MDMA, 3,4-methylenedioxymethamphetamine; SDS, Sheehan Disability Scale.

a Benzodiazepines, z-hypnotics and ADHD medication not included.

b Mianserin, mirtazapine, bupropion or vortioxetine.

### Primary outcome

As displayed in Table 2, the mean MADRS score at the primary outcome visit (V13, i.e. approximately 2 months after the second MDMA dosing session) was 9.7 (s.d. 10.0), giving a mean of −19.3 (s.d. 8.3). Confidence interval for change in MADRS (bootstrap with 4000 resamples of the mean change) was −14.9 to −23.7. The reduction in MADRS was statistically significant (*z* = −3.1, *P* < 0.001; see Supplementary Figs 2, 3 and 6 for details). Among the 9 participants without comorbid PTSD, the mean change in MADRS score from baseline to post treatment was −18.1 (s.d. 8.8), similar to the full sample.

**Table 2:** Overall summary of primary, secondary and selected exploratory efficacy outcomes

	Baseline^b^ mean (s.d.)	Outcome^c^ mean (s.d.)	Difference mean (95% CI)	*Z*-value	*P*-value
MADRS (primary outcome)^a^	29.6 (4.9)	9.7 (10.0)	−19.3 (−14.9, -23.7)	−3.06	<0.001
SDS total score (secondary outcome)	21.3 (6.1)	9.7 (9.2)	−11.7 (−7.6, −15.8)	−3.02	0.001
SDS days lost	2.5 (2.2)	1.0 (2.1)	−2.0 (−0.8, −3.2)	−2.41	0.031
SDS underproductive days	6.1 (1.8)	2.4 (3.0)	−4.1 (−2.2, −5.9)	−2.57	0.008
BDI-II	33.5 (9.6)	12.0 (14.8)	−21.5 (−14.0, −29.0)	−2.98	0.001
GAD-7	13.8 (4.2)	6.0 (5.4)	−9.0 (−6.0, −12.0)	−2.89	0.002
PCL-5	36.4 (17.0)	25.4 (19.7)	−11.0 (−2.4, −19.6)	−2.12	0.032
BIS	28.9 (7.5)	12.6 (10.5)	−16.3 (−9.5, −23.1)	−2.79	0.003
AUDIT	3.3 (2.5)	3.3 (2.5)	−0.1 (−0.8, 1.0)	0.16	0.981
DUDIT	0.6 (0.9)	0.1 (0.3)	−0.5 (−1.0, 0)	−1.47	0.188

MADRS, Montgomery–Asberg Depression rating scale; SDS, Sheehan Disability Scale; BDI-II, Beck’s Depression Inventory-II; GAD-7, Generalised Anxiety Disorder-7; PCL-5, the Post-Traumatic Stress Disorder Checklist for DSM-5; BIS, Bergen Insomnia Scale; AUDIT, Alcohol Use Disorders Identification Test; DUDIT, Drug Use Disorder Identification Test.

a All outcomes are exploratory unless otherwise noted.

b Visit 3 (V3) for MADRS and SDS, screening for AUDIT and DUDIT and V4 for other outcome measures.

c V13 for MADRS and SDS, V14 for other outcome measures.

### Secondary outcome

Mean SDS total score at the outcome visit was 9.7 (s.d. 9.2), resulting in a mean change of −11.7 (s.d. 7.6), as shown in Table 2. The 95% CI for change in SDS total score, based on bootstrap resampling, was 7.6−15.8. The reduction in SDS scores was statistically significant (*z* = −3.0, *P* = 0.001; see Supplementary Figs 2, 3 and 6).

### Exploratory analysis

At the primary outcome visit, 9 out of 12 participants (75.0%) were classified as responders, showing a reduction of ≥50% in MADRS scores. Similarly, 9 out of 12 (75.0%) were classified as remitters, with MADRS scores of ≤12.

As demonstrated in Table 2, there was a statistically significant improvement between baseline and post treatment for the selected exploratory outcomes BIS, GAD-7, BDI and PCL-5.

**Fig. 2:**
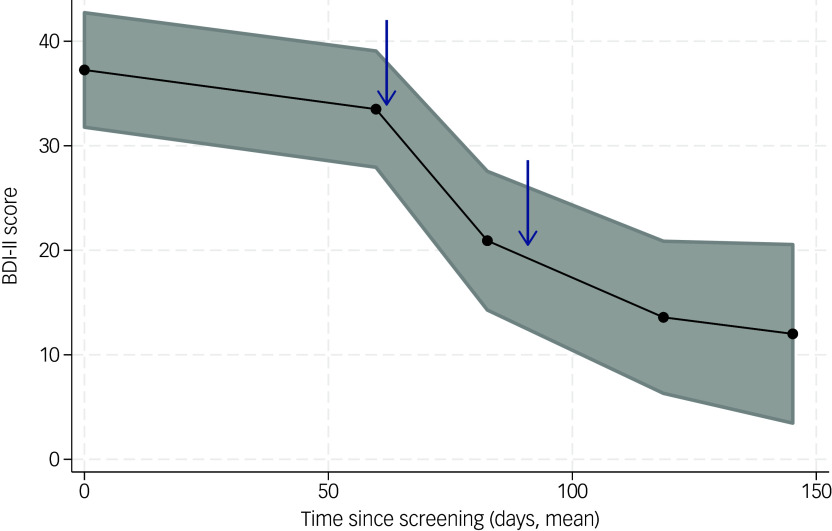
The trajectory of Beck’s Depression Inventory (BDI) scores. Black line represents the means, and grey shaded area ±2 s.e. Dots represent different visits (screening, visit 4 (V4), V8, V12 and V14). 3,4-methylenedioxymethamphetamine dosing occurred at V5 and V9, marked by blue arrows.

The trajectory of mean BDI-II scores and ±2 standard errors are shown in Fig. 2. The mixed-effects model estimated a regression coefficient of −0.19 (95% CI −0.24 to −0.15, *z* = −8.32, *P* < 0.0001), indicating that for each consecutive day after the first screening visit, BDI-II score decreased by 0.19 points. The non-linear progress depicted in Fig. 2, starting with a slight decline until the first visit and followed by a parabola-like progression, was also assessed in the subsequent mixed-effects model. This did, however, find only a linear decline throughout the period of the trial. This apparent difference is explained by considerable variation in the timing of visits among participants. The time points for the visits given in Fig. 2 are averaged over all participants.

### Safety

As shown in Supplementary Table 2, ten participants had experienced at least one adverse event. A total of 46 adverse events occurred, and all except one (suicidal ideation deterioration before administration of study drug) were classified as TEAEs. Of these, 15 were of moderate severity while 31 were mild. The 3 non-responders experienced a total of 25 adverse events (13 of moderate severity), while the 9 responders reported 21 adverse events (2 of moderate severity). There were no serious or severe adverse events, or AESIs. There were no discontinuations of MDMA or MDMA-AT, no instances of serious suicidal ideation or behaviour nor any hospitalisations. The most commonly reported TEAEs were headache, increase in suicidal ideation and jaw muscle tightness. Ten of the TEAEs required psychotherapeutic support at scheduled study visits. Three participants required a total of seven extra visits (two, two and three, respectively) on-site or by telephone beyond scheduled study visits to give support and reassurance, and to facilitate further processing of emergent material and ensure participant safety. A full list of adverse events is given in Supplementary Table 3. The duration of adverse events was usually transient: mean duration was 7.3 days (s.d. 17.6), but here a few outliers contributed to a highly right-skewed distribution (skewness 3.8, median 1.0). Of 46 adverse events, 28 lasted 1 day only whereas 39 had resolved within 1 week. The mean duration of adverse events was 11.6 days (s.d. 23.1) in the non-responder group compared with 2.1 days (s.d. 2.4) in the responder group.

Two participants required psychotropic medication as a result of TEAEs. Within the 2-day window following MDMA dosing sessions, both received benzodiazepines and zopiclone to manage anxiety and insomnia. Additionally, one of these participants received benzodiazepine during the treatment period but outside of the 2-day window. Eight of the TEAEs required non-prescription drugs, specifically paracetamol for headaches.

Aside from the treatment of TEAEs, 6 participants were administered psychotropic medications during the treatment period (between V5 and V12) for medical or psychiatric conditions, in accordance with the study team and the protocol. Among these, two participants resumed lisdexamfetamine for attention-deficit hyperactivity disorder while another participant received codeine for a rib fracture unrelated to the study. The remaining participants were treated with hydroxyzine, zopiclone, alimemazine or oxazepam for comorbid anxiety or insomnia.

There were no instances of serious suicidal ideation, defined as a C-SSRS score of 4 or 5, nor were there any occurrences of suicidal behaviour. One participant experienced an increase in suicidal ideation score throughout the study period. However, six participants (12 adverse events) exhibited a worsening in the intensity of their suicidal ideation score. There were no reports of suicidal ideation during the MDMA dosing sessions. Neither mean suicidal ideation score nor mean intensity of suicidal ideation score exceeded the mean baseline score (over the past 6 months) measured during screening. The mean suicidal ideation score at post treatment for participants without PTSD was 0.6 (s.d. 1.0), with a mean suicidal ideation intensity rating of 4.1 (s.d. 6.2). For the overall sample, these values were 0.4 (s.d. 0.9) and 3.1 (s.d. 5.6), respectively. Supplementary Fig. 4 illustrates the trajectory and intensity of suicidal ideation, respectively, throughout the study visits.

There were no adverse events related to cardiovascular events or cardiac arrhythmias. Transient elevations in blood pressure, heart rate and body temperature were observed during MDMA dosing sessions, as indicated in Supplementary Fig. 5.

There were no reports of adverse events related to MDMA use outside the study. As shown in Table 2, there were no statistically significant changes in the risk of hazardous alcohol or substance use as measured by AUDIT and DUDIT, respectively.

## Discussion

In this small, open-label, proof-of-concept trial, we explored MDMA-AT as a potential novel treatment for MDD. With careful screening, assessment and psychotherapy throughout the study, we demonstrated that MDMA-AT can be safely administered to participants with MDD. All adverse events were expected, most being mild and transient, and no serious adverse events or AESIs were reported. To manage adverse events, two participants were given benzodiazepines and zopiclone for anxiety and insomnia while three received extra psychotherapy sessions. The frequency, severity and duration of adverse events varied according to respondent status. Recruitment and retention rates were adequate. Thus, the study successfully met its primary objectives of feasibility and safety. Furthermore, we observed statistically significant improvements in both depression and functional impairment.

Consistent with previous studies of MDMA-AT for PTSD (e.g. ref.^17^), we demonstrated both statistically and clinically significant reductions in the primary and secondary outcome measures of depression and functional impairment. Similar to PTSD studies, we observed a high proportion of both responders and remitters. In the present study on MDD, 9 out of 12 participants were classified as both responders and remitters based on MADRS scores, comparable to the loss of PTSD diagnosis in 67 and 71% of participants in the MDMA groups of the two respective phase 3 studies.^17,21^ Unlike previous phase 2 and 3 studies that relied on the self-reported measure BDI-II, we utilised MADRS as a clinician-rated assessment of depression. Similarly to the PTSD studies, we also observed a statistically and clinically significant reduction in PTSD symptoms, exceeding a 10-point decrease on PCL-5.^22^ Although only three participants met the criteria for PTSD diagnosis at baseline, most addressed traumatic autobiographical material, attachment injuries and/or relational trauma, which appeared to contribute to the observed reduction in depressive symptoms. Although the study was not powered to support subgroup analyses, preliminary comparisons indicate that treatment responses and safety profiles were broadly comparable between participants with and without PTSD.

The most common adverse events in this study differed from those in the PTSD phase 3 trials MAPP1 and MAPP2.^17,21^ Generally, fewer adverse events were observed here, with dry mouth frequency similar to that in MAPP2. However, decreased appetite (16.7 *v*. 52.2% in MAPP1) and anxiety (25.0 *v*. 32.6% in MAPP1) were less frequent. Headache (58.4 *v*. 71.7% in MAPP1) and suicidal ideation (33.3 *v*. 45.7% in MAPP1) were also reported less often. These differences may reflect actual variations or factors such as underreporting. As in PTSD studies,^17,21^ group level of suicidality in the present study never exceeded that of baseline, although there were variations at the individual level. Jaw muscle tightness was more common in this study than jaw pain in MAPP1, possibly due to adverse event labelling differences.

The current sample was predominantly well educated, with a high level of functional impairment at baseline, and primarily consisted of individuals with moderate severity MDD. The sample population was predominantly of White ethnicity, so the findings may not be generalisable to Black and minority ethnic participants. While a standardised measure of treatment-resistant depression was not used for eligibility, the level of treatment resistance, particularly to psychotherapy, was considerable. While speculative, it is possible that this level of prior psychotherapeutic experience was beneficial during the MDMA-AT sessions, potentially contributing to the positive outcome. It is worth noting that, because nearly all participants were recruited through self-referral, similar to earlier reports,^17,21^ this may have resulted in a sample that is less representative of the general MDD population. Additionally, the self-referred group may have had elevated expectations regarding the effectiveness of MDMA-assisted therapy, potentially exaggerating the pre–post differences in outcome measures.

We implemented a comprehensive psychotherapeutic regimen in delivering MDMA-AT for MDD. Prior training and experience from a similar MDMA-AT study was important, and team discussions to manage transference, countertransference and other challenges all proved valuable. The observations of the psychotherapy team suggest that the inner-directed psychotherapeutic approach supports the processing and analysis of spontaneously emerging psychotherapeutic material during MDMA sessions for MDD, as it does for PTSD. The phase 3 trials for PTSD indicated added benefit from a third session.^16,17^ Several participants in the present study spontaneously expressed the opinion that a third session would have been beneficial. Future research should explore the optimal number of sessions and consider a flexible dosing approach, allowing for additional sessions when indicated.

We provided psychotherapy to manage psychological distress and related TEAEs (such as increased anxiety) in scheduled and extra visits on-site or by telephone. This suggests that integrative sessions, follow-up telephone calls and a flexible protocol allowing extra visits improve the benefits but also ensure safety, in line with previous findings.^23^ Given the absence of suicidal ideation at the end of dosing sessions, the current study suggests that an overnight hospital stay may be less critical in MDMA-AT for MDD, which is also supported by recent data.^21^

The current study has some limitations. The lack of a control group prevented us from determining whether the therapeutic response was attributable to the MDMA-AT provided, expectancy effects or natural fluctuations in depression severity over time. Although we used MADRS raters who were not part of the therapy team and were unaware of participants’ depression history or present condition, they were neither completely independent nor blinded. As a result, a potential rater bias may have inflated pre–post difference in MADRS scores. Future studies should include blinded and independent raters, e.g. as in refs.^17,21^ Because adverse events were reported to the therapy team, there is a possibility of underreporting due to participants’ relationship to the therapists. It would have been preferable for adverse events to be assessed by an independent adverse event assessor. We did not assess adherence to the MDMA-AT therapy manual,^24^ which may have resulted in deviations from the manual and variations in the therapeutic support provided. However, the therapists had their adherence rated in a previous MDMA trial,^20^ with supervision from an experienced MDMA-AT supervisor. Finally, in the mixed-effects model, the time intervals between visits varied considerably between patients.

Although this small, uncontrolled trial cannot draw conclusions about the efficacy of MDMA-AT for MDD, the findings suggest that MDMA-AT has potential as a treatment for MDD, and supports future randomised controlled trials (RCTs). If demonstrated to be effective and safe in RCTs, MDMA-AT could represent a significant advancement in the treatment of MDD, offering an integrated approach where the drug is used several times to catalyse psychotherapy rather than being administered daily as is the case with antidepressants.

In conclusion, this is the first trial of MDMA-AT for a primary diagnosis of MDD, successfully meeting the primary objectives of safety and feasibility. The statistically and clinically significant reductions in depressive symptoms and functional impairment following treatment warrant further investigation in randomised trials.

## Supporting information

Kvam et al. supplementary material 1 Kvam et al. supplementary material

Kvam et al. supplementary material 2 Kvam et al. supplementary material

Kvam et al. supplementary material 3 Kvam et al. supplementary material

Kvam et al. supplementary material 4 Kvam et al. supplementary material

Kvam et al. supplementary material 5 Kvam et al. supplementary material

Kvam et al. supplementary material 6 Kvam et al. supplementary material

Kvam et al. supplementary material 7 Kvam et al. supplementary material

Kvam et al. supplementary material 8 Kvam et al. supplementary material

Kvam et al. supplementary material 9 Kvam et al. supplementary material

Kvam et al. supplementary material 10 Kvam et al. supplementary material

Kvam et al. supplementary material 11 Kvam et al. supplementary material

## Data Availability

The data that support the findings of this study are available on reasonable request from the corresponding author, T.-M.K. However, the data are not publicly accessible due to restrictions related to the inclusion of information that could compromise the privacy of research participants.

## Supplementary material

The supplementary material is available online at https://doi.org/10.1192/bjp.2025.10320
