# MDMA-assisted therapy as a treatment for major depressive disorder: proof of principle study – CORRIGENDUM

**DOI:** 10.1192/bjp.2026.10579

**Published:** 2026-04

**Authors:** Tor-Morten Kvam, Ivar W. Goksøyr, Justyna Rog, Inger-Tove Jentoft van de Vooren, Lowan Han Stewart, Ingrid Autran, Mark Berthold-Losleben, Lynn Mørch-Johnsen, René Holst, Ingmar Clausen, Ole A. Andreassen

**Affiliations:** Institute of Clinical Medicine, University of Oslo, Norway; Nordre Østfold DPS, Østfold Hospital Trust, Grålum, Norway; Oslo University Hospital, Oslo, Norway; Department of Mental Health, Norwegian University of Science and Technology, Trondheim, Norway; Department of Psychiatry, Østfold Hospital Trust, Grålum, Norway; Department of Clinical Research, Østfold Hospital Trust, Grålum, Norway

Since this article’s publication, the authors have discovered and reported a minor error after analysis of follow-up data relating to one participant’s post-treatment Montgomery–Åsberg Depression Rating Scale (MADRS) score.

The error does not affect the reported number of responders or remitters or the MADRS scores among the nine participants without comorbid post-traumatic stress disorder. The error does not alter any of the study’s interpretation of the results, or the conclusions.

The corrected table is below:

**Table 2:** Overall summary of primary, secondary and selected exploratory efficacy outcomes

	Baseline^b^ mean (s.d.)	Outcome^c^ mean (s.d.)	Difference mean (95% CI)	*Z*-value	*P*-value
MADRS (primary outcome)^a^	29.6 (4.9)	10.3 (8.3)	−19.3 (−14.9, −23.7)	−3.06	<0.001
SDS Total Score (secondary outcome)	21.3 (6.1)	9.7 (9.2)	−11.7 (−7.6, −15.8)	−3.02	0.001
SDS Days lost	2.5 (2.2)	1.0 (2.1)	−2.0 (−0.8, −3.2)	−2.41	0.031
SDS underproductive days	6.1 (1.8)	2.4 (3.0)	−4.1 (−2.2, −5.9)	−2.57	0.008
BDI-II	33.5 (9.6)	12.0 (14.8)	−21.5 (−14.0, −29.0)	−2.98	0.001
GAD-7	13.8 (4.2)	6.0 (5.4)	−9.0 (−6.0, −12.0)	−2.89	0.002
PCL-5	36.4 (17.0)	25.4 (19.7)	−11.0 (−2.4, −19.6)	−2.12	0.032
BIS	28.9 (7.5)	12.6 (10.5)	−16.3 (−9.5, −23.1)	−2.79	0.003
AUDIT	3.3 (2.5)	3.3 (2.5)	−0.1 (−0.8, 1.0)	0.16	0.981
DUDIT	0.6 (0.9)	0.1 (0.3)	−0.5 (−1.0, 0.0)	−1.47	0.188

MADRS, Montgomery–Asberg Depression rating scale; SDS, Sheehan Disability Scale; BDI-II, Beck’s Depression Inventory-II; GAD-7, Generalised Anxiety Disorder-7; PCL-5, the Post-Traumatic Stress Disorder Checklist for DSM-5; BIS, Bergen Insomnia Scale; AUDIT, Alcohol Use Disorders Identification Test; DUDIT, Drug Use Disorder Identification Test.

a. All outcomes are exploratory unless otherwise noted.

b. Visit 3 (V3) for MADRS and SDS, screening for AUDIT and DUDIT and V4 for other outcome measures.

c. V13 for MADRS and SDS, V14 for other outcome measures.

The same corrected values should also be applied to the ‘Primary outcome’ section of the paper, on page 785.
