# Supplementary material for: MDMA-assisted therapy as a treatment for major depressive disorder: proof of principle study
Source: Br J Psychiatry. 2025 Jul 11;227(5):783–9. doi: 10.1192/bjp.2025.10320 (PMC12550655; doi:10.1192/bjp.2025.10320)
Supplement: Kvam et al. supplementary material 1 — Kvam et al. supplementary material [file S0007125025103206sup001.pdf]

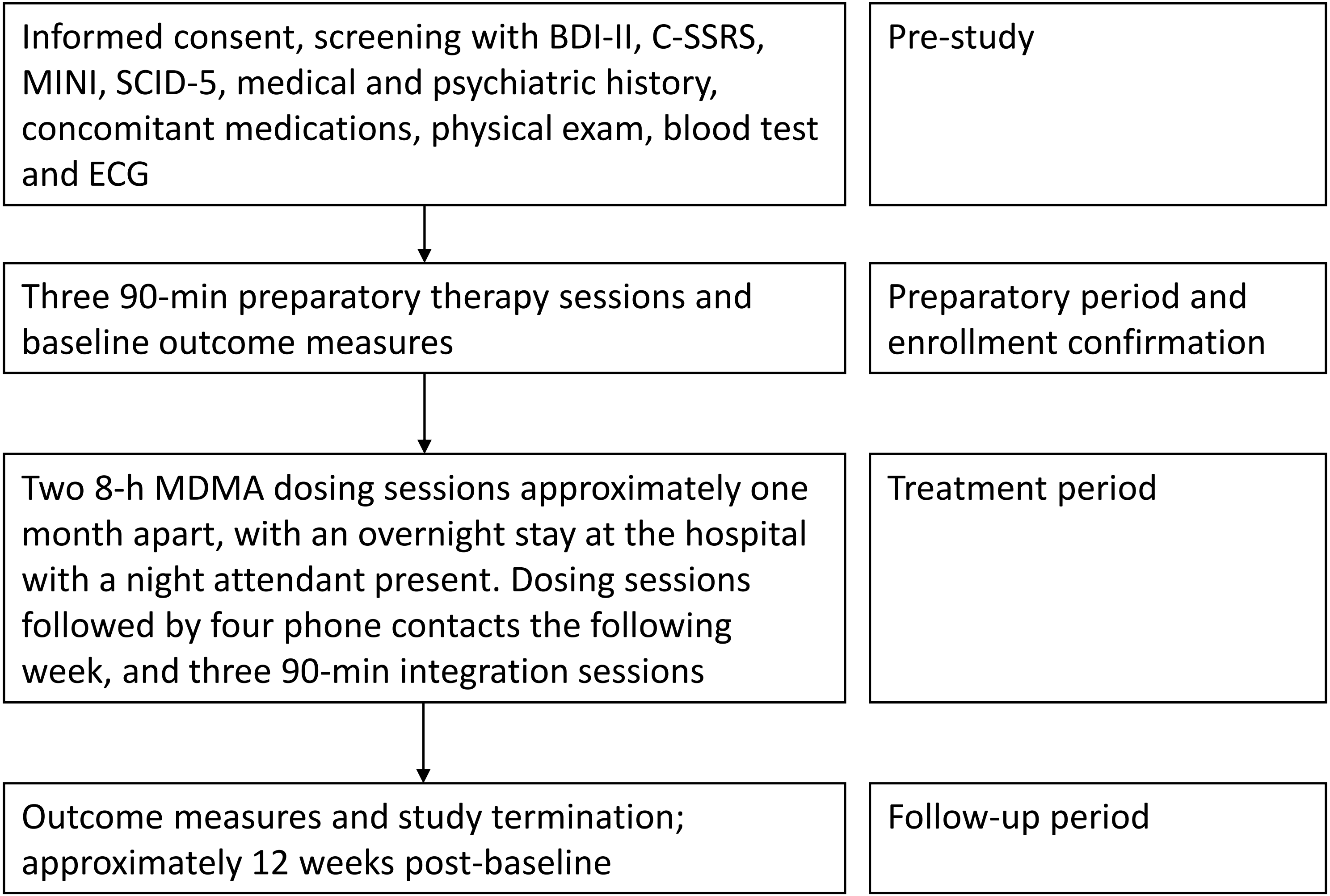
Informed consent, screening with BDI-II, C-SSRS, MINI, SCID-5, medical and psychiatric history, concomitant medications, physical exam, blood test and ECG
Pre-study
Three 90-min preparatory therapy sessions and baseline outcome measures
Preparatory period and enrollment confirmation
Two 8-h MDMA dosing sessions approximately one month apart, with an overnight stay at the hospital with a night attendant present. Dosing sessions followed by four phone contacts the following week, and three 90-min integration sessions
Treatment period
Outcome measures and study termination; approximately 12 weeks post-baseline
Follow-up period
