# Supplementary figures and images for: MDMA-assisted therapy as a treatment for major depressive disorder: proof of principle study
Source: Br J Psychiatry. 2025 Jul 11;227(5):783–9. doi: 10.1192/bjp.2025.10320 (PMC12550655; doi:10.1192/bjp.2025.10320)

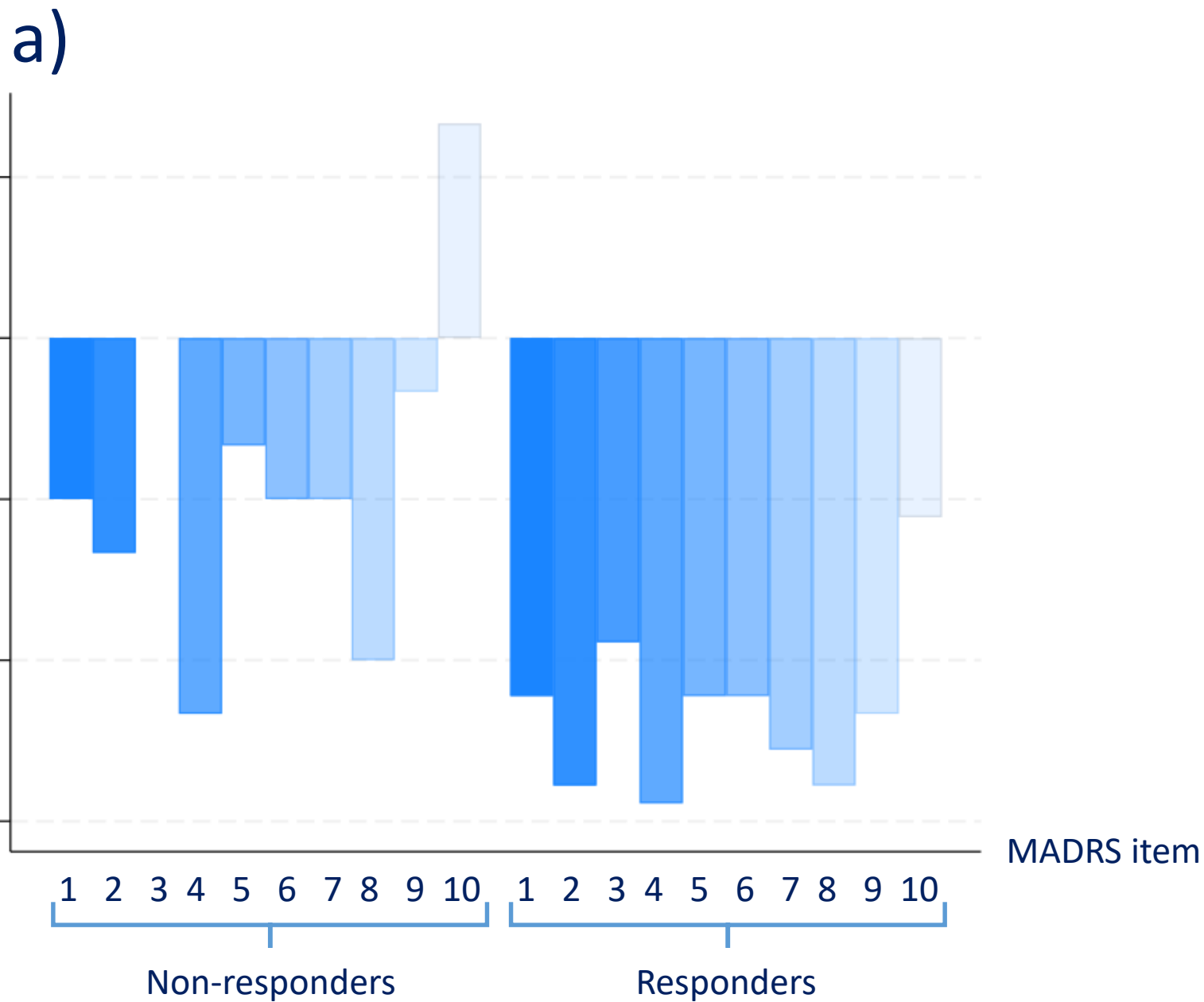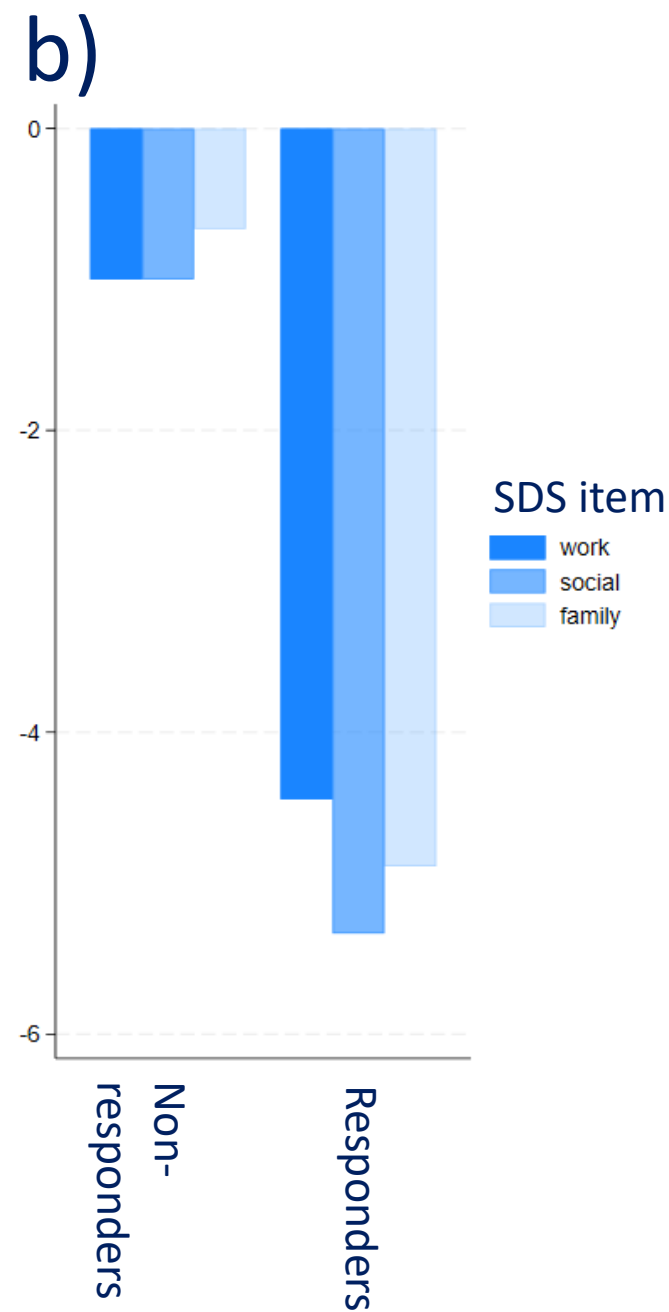

Supplement: Kvam et al. supplementary material 2 — Kvam et al. supplementary material [file S0007125025103206sup002.pdf]

## Slide 1
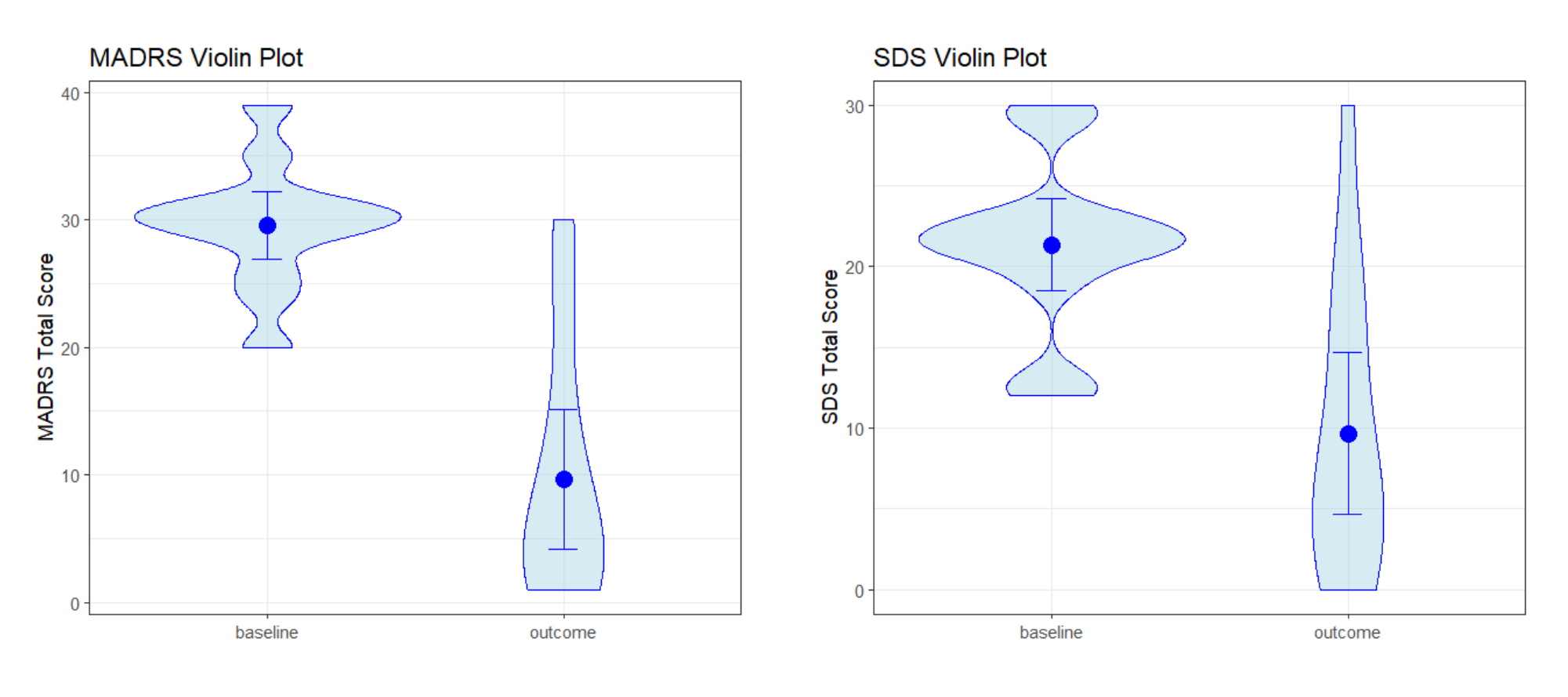

Supplement: Kvam et al. supplementary material 3 — Kvam et al. supplementary material [file S0007125025103206sup003.pptx]

a) Suicidal ideation

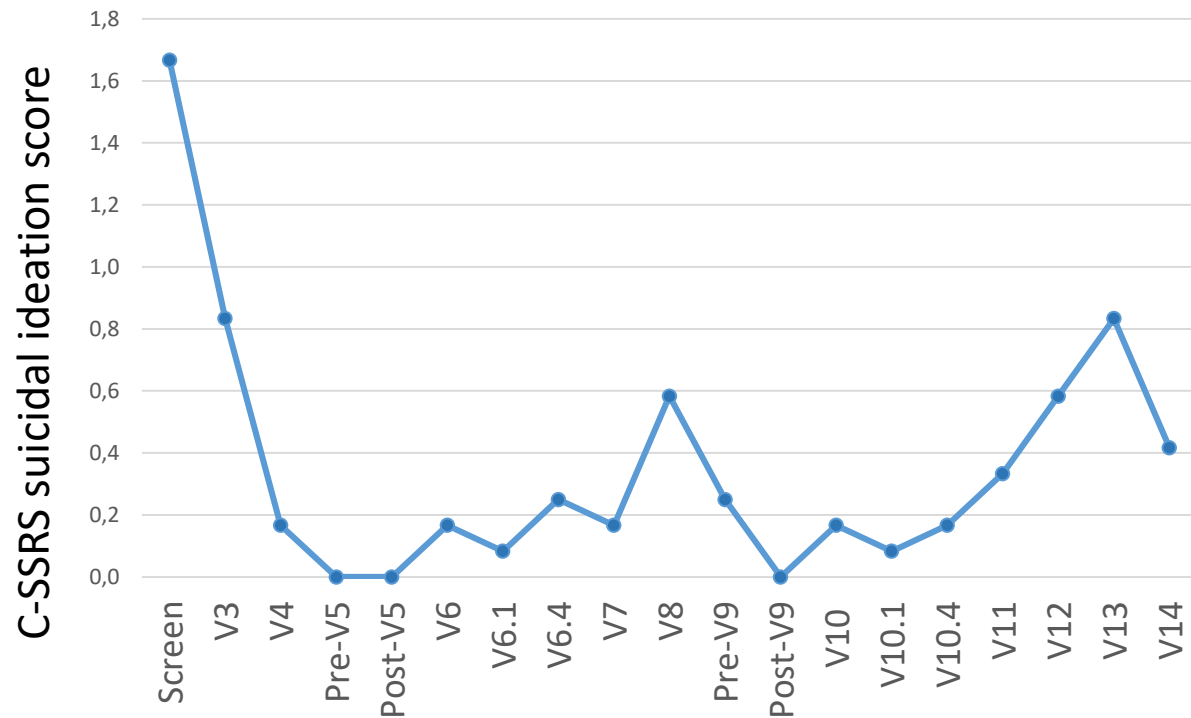

b) Intensity of ideation

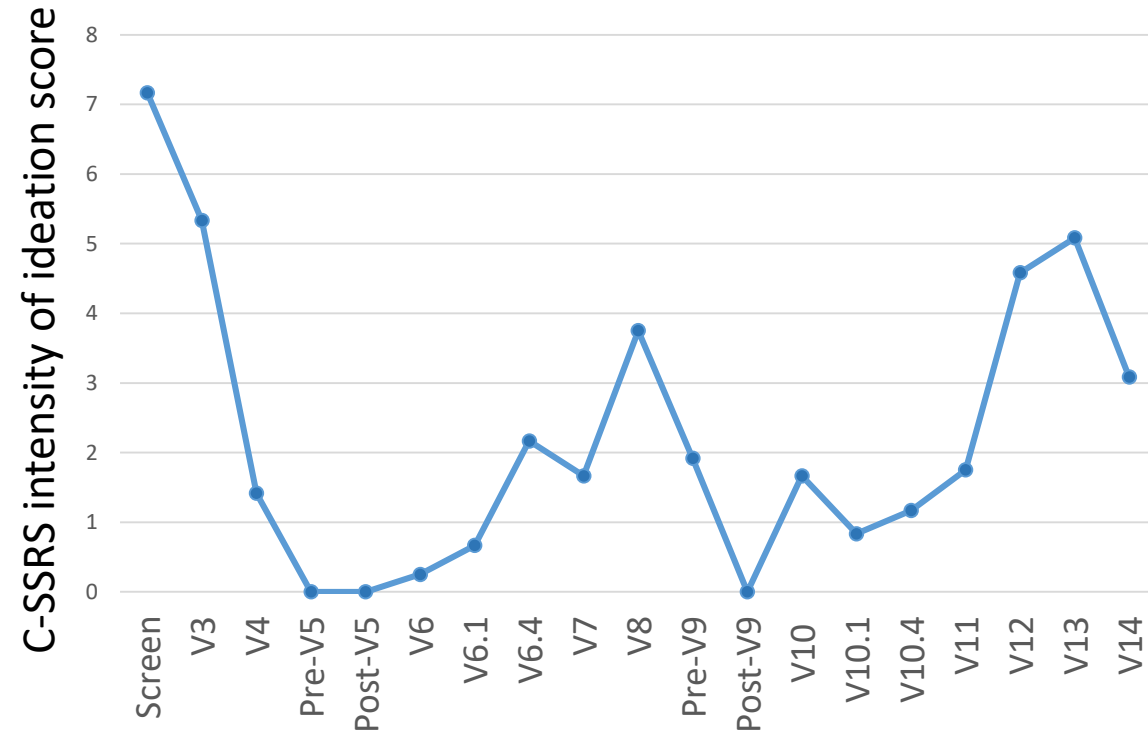

Supplement: Kvam et al. supplementary material 4 — Kvam et al. supplementary material [file S0007125025103206sup004.pdf]

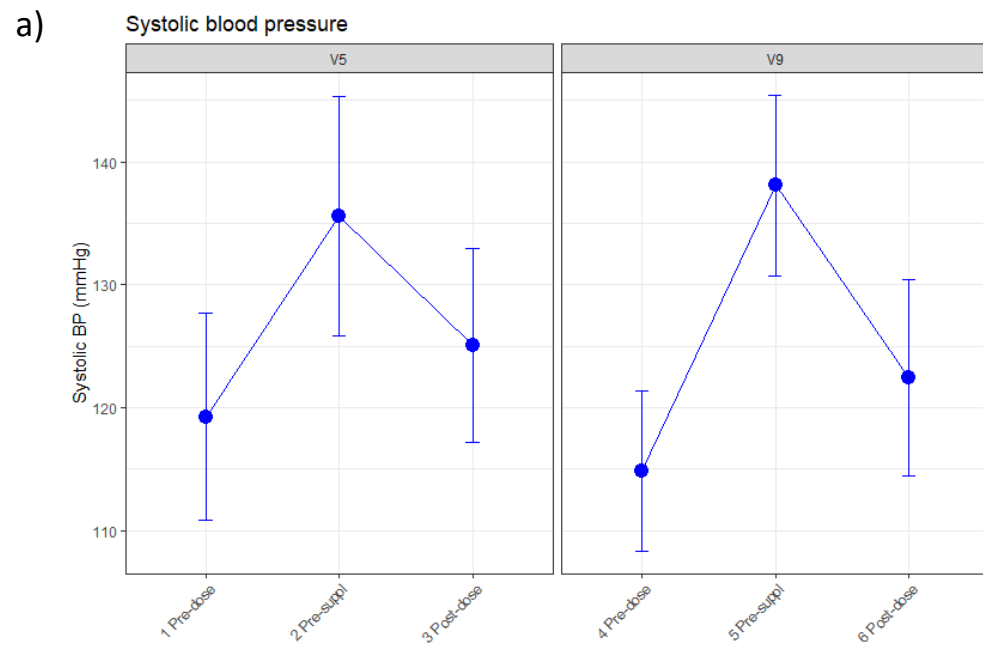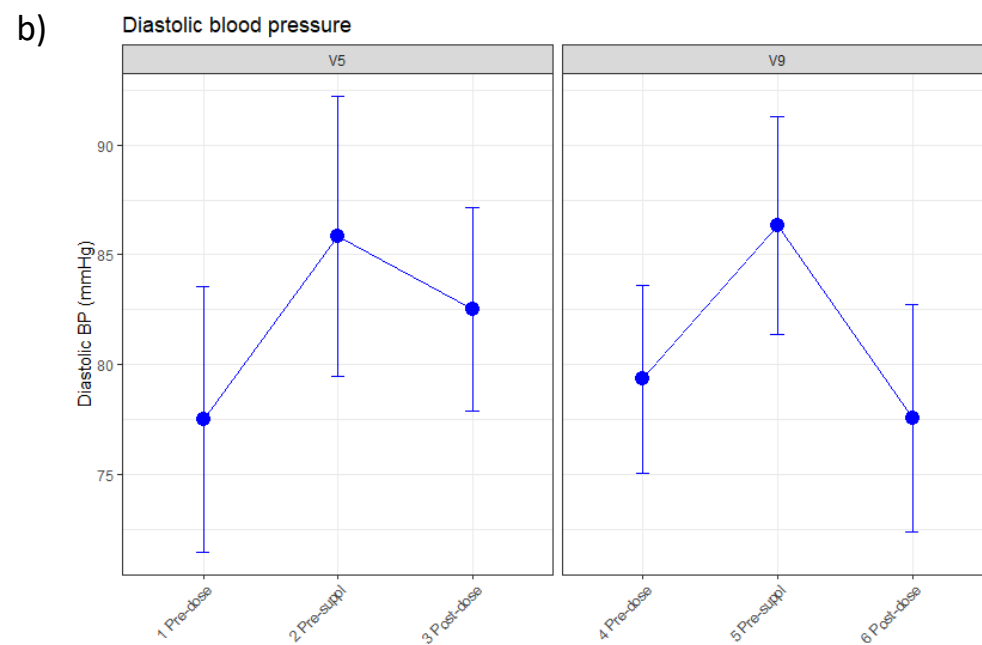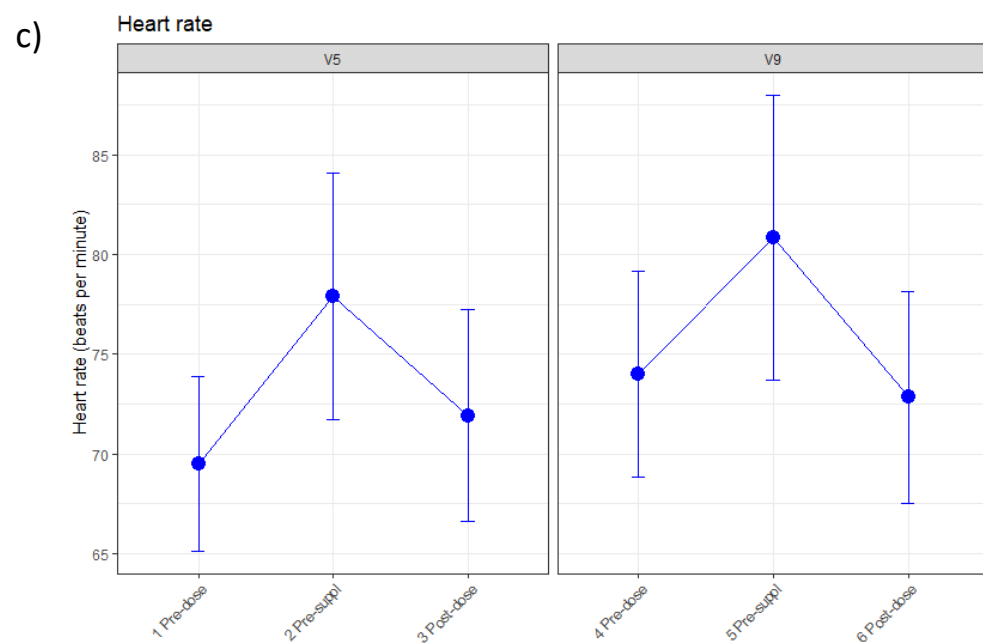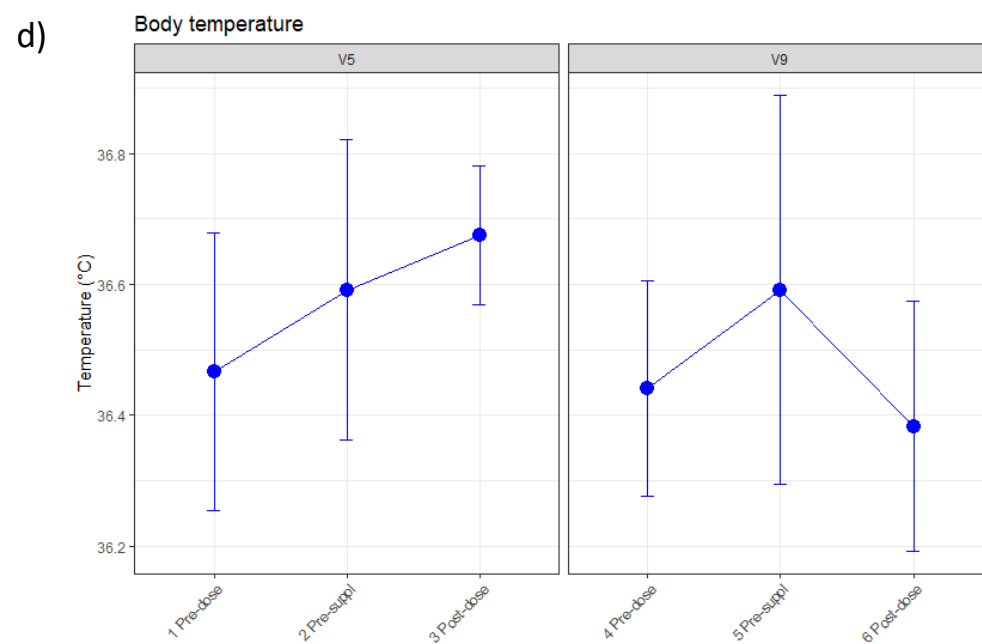

Supplement: Kvam et al. supplementary material 5 — Kvam et al. supplementary material [file S0007125025103206sup005.pdf]

## Slide 1
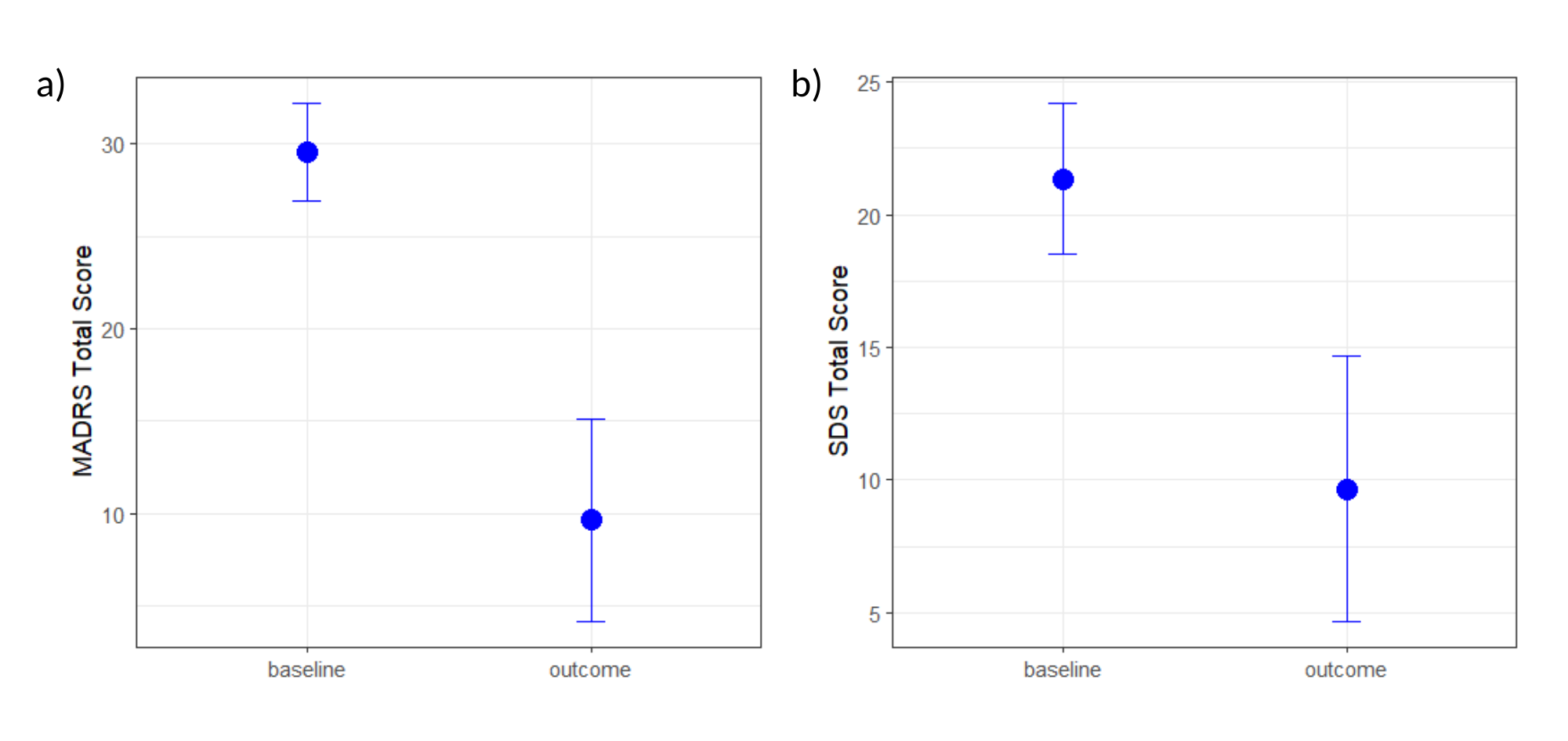

b)
a)

Supplement: Kvam et al. supplementary material 6 — Kvam et al. supplementary material [file S0007125025103206sup006.pptx]
