# Supplementary material for: MDMA-assisted therapy as a treatment for major depressive disorder: proof of principle study
Source: Br J Psychiatry. 2025 Jul 11;227(5):783–9. doi: 10.1192/bjp.2025.10320 (PMC12550655; doi:10.1192/bjp.2025.10320)
Supplement: Kvam et al. supplementary material 7 — Kvam et al. supplementary material [file S0007125025103206sup007.docx]

[insert Supplementary Figure 1 here]

**Supplementary Figure 1 Study Structure Overview.** BDI-II: Beck’s Depression Inventory II. MINI: Mini International Neuropsychiatric Interview. C-SSRS: Columbia-Suicide Severity Rating Scale. SCID-5: Structured Clinical Interview for DSM-5

[insert Supplementary Figure 2 here]

**Supplementary Figure 2. Change of single items from baseline to the outcome visit.** a): Montgomery-Asberg Depression Rating Scale (MADRS) and b): Sheehan Disability Scale (SDS). The MADRS single items ranges from 0 to 6, higher scores reflecting greater depression severity. SDS single items ranges from 0 to 10, higher scores reflecting greater functional impairment.

[insert Supplementary Figure 3 here]

**Supplemental figure 3. Violin plots of a) Montgomery-Asberg Depression Rating Scale (MADRS) and b) Sheehan Disability Scale (SDS) total scores at baseline and outcome visits.** The total score on the MADRS ranges from 0 to 60, with higher scores reflecting greater depression severity. The total score on the SDS ranges from 0 to 30, with higher scores reflecting greater functional disability. In the violin plot, the width of each curve represents the approximate frequency of data points. Dots indicate means and error bars indicate 95% confidence intervals.

[insert Supplementary Figure 4 here]

**Supplemental figure 4 The trajectory of mean a) suicidal ideation; and b) intensity of the ideation as measured by the Columbia-Suicide Severity Rating Scale (C-SSRS).** The C-SSRS were measured on every study visit except V1 and V2. There were two measures on MDMA dosing days (V5 and V9), i.e. before and after the session. V6.1, V6.4, V10.1, and V10.4 were follow-up phone calls conducted after the first integration session following each MDMA dosing session.

[insert Supplementary Figure 5 here]

**Supplementary Figure 5 Vital signs.** a) Systolic blood pressure; b) Diastolic blood pressure; c) Heart rate; and d) Body temperature. Panels show vital signs in Visit 5 and 9, respectively. Dots represent means and error bars represent 95% CI. The time points for the vital signs are as follows: pre-dose = before administration of the initial dose; pre-suppl = before administration of the supplemental dose (1.5-2 hours after the initial dose); and post-dose = at the end of the day-long dosing session (6-8 hours after the initial dose).

[insert Supplementary 6 here]

**Supplementary Figure 6 a) MADRS and b) SDS total scores at baseline and outcome visits.** a) The total score on the Montgomery–Åsberg Depression Rating Scale (MADRS) ranges from 0 to 60, with higher scores reflecting greater depression severity. Dots indicate means and error bars indicate 95% confidence intervals. b) The Sheehan Disability Scale (SDS) ranges from 0 to 30, with higher scores reflecting greater functional impairment.
