# Supplementary material for: MDMA-assisted therapy as a treatment for major depressive disorder: proof of principle study
Source: Br J Psychiatry. 2025 Jul 11;227(5):783–9. doi: 10.1192/bjp.2025.10320 (PMC12550655; doi:10.1192/bjp.2025.10320)
Supplement: Kvam et al. supplementary material 8 — Kvam et al. supplementary material [file S0007125025103206sup008.docx]

Supplementary table 1. Dose regimen of MDMA.

| **Dosing Session** | **Initial Dose** | **Supplemental Dose** | **Min-Max Cumulative Dose** |
| --- | --- | --- | --- |
| 1 | 80 mg | 40 mg | 80 mg to 120 mg |
| 2 | 80 or 120 mg | 40 or 60 mg | 80 mg to 180 mg |
| **Total Cumulative Dose** | | | 160 mg to 300 mg |
