# Supplementary material for: MDMA-assisted therapy as a treatment for major depressive disorder: proof of principle study
Source: Br J Psychiatry. 2025 Jul 11;227(5):783–9. doi: 10.1192/bjp.2025.10320 (PMC12550655; doi:10.1192/bjp.2025.10320)
Supplement: Kvam et al. supplementary material 9 — Kvam et al. supplementary material [file S0007125025103206sup009.docx]

| Summary of TEAEs | No. |  | % |
| --- | --- | --- | --- |
| Participants with ≥1 TEAE | 9 |  | 75.0 |
| Participants with ≥1 severe TEAE | 0 |  | 0.0 |
| Participants with ≥1 serious ADVERSE EVENT | 0 |  | 0.0 |
| Participants with ≥1 TEAE leading to study discontinuation | 0 |  | 0.0 |
| Participants with ≥1 AESI | 0 |  | 0.0 |
| Most common TEAS^A^ |  |  |  |
| Headache | 7 |  | 58.3 |
| Deterioration of suicidal ideation | 4 |  | 33.3 |
| Jaw muscle tightness | 4 |  | 33.3 |
| Anxiety | 3 |  | 25.0 |
| Decreased appetite | 2 |  | 16.7 |
| Dry mouth | 2 |  | 16.7 |

Supplementary table 2 | Adverse events occurring during treatment. A) occurring in ≥2 individuals. AESI: adverse event of special interest.
