# Supplementary material for: MDMA-assisted therapy as a treatment for major depressive disorder: proof of principle study
Source: Br J Psychiatry. 2025 Jul 11;227(5):783–9. doi: 10.1192/bjp.2025.10320 (PMC12550655; doi:10.1192/bjp.2025.10320)
Supplement: Kvam et al. supplementary material 10 — Kvam et al. supplementary material [file S0007125025103206sup010.docx]

Supplementary table 3. A complete list of adverse events (AEs) in the study. SAE: serious adverse event. AESI: adverse event of special interest. TEAE: treatment-emergent adverse event.

| AE # (total) | AE # (per participant) | Description | SAE | AESI | TEAE | Present before IMP administration | Duration (days) | Severity | Consequence for the trial | Treatment provided | Outcome |
| --- | --- | --- | --- | --- | --- | --- | --- | --- | --- | --- | --- |
| 1 | 1 | Headache | No | No | Yes | No | 1 | Moderate | None | Non-prescription drug | Complete recovery/return to baseline condition |
| 2 | 2 | Jaw muscle tightness | No | No | Yes | No | 1 | Mild | None | None | Complete recovery/return to baseline condition |
| 3 | 3 | Tinnitus | No | No | Yes | No | 7 | Mild | None | None | Complete recovery/return to baseline condition |
| 4 | 4 | Drowsiness | No | No | Yes | No | 1 | Mild | None | None | Complete recovery/return to baseline condition |
| 5 | 5 | Headache | No | No | Yes | No | 1 | Moderate | None | Non-prescription drug | Complete recovery/return to baseline condition |
| 6 | 6 | Jaw muscle tightness | No | No | Yes | No | 1 | Moderate | None | None | Complete recovery/return to baseline condition |
| 7 | 7 | Anxiety | No | No | Yes | No | ≥35 days, continuing at least until the study termination | Moderate | None | None | Persisting, stabile |
| 8 | 8 | Deterioration of suicidal ideation | No | No | Yes | No | 14 | Moderate | None | None | Complete recovery/return to baseline condition |
| 9 | 9 | Deterioration of suicidal ideation | No | No | Yes | No | 15 | Moderate | None | Psychotherapy | Complete recovery/return to baseline condition |
| 10 | 10 | Deterioration of suicidal ideation | No | No | Yes | No | 1 | Moderate | None | None | Complete recovery/return to baseline condition |
| 11 | 1 | Derealization | No | No | Yes | No | 7 | Moderate | None | None | Complete recovery/return to baseline condition |
| 12 | 2 | Loss of appetite | No | No | Yes | No | 1 | Mild | None | None | Complete recovery/return to baseline condition |
| 13 | 1 | Headache | No | No | Yes | No | 1 | Mild | None | None | Complete recovery/return to baseline condition |
| 14 | 2 | Headache | No | No | Yes | No | 2 | Mild | None | Non-prescription drug | Complete recovery/return to baseline condition |
| 15 | 3 | Teeth chattering | No | No | Yes | No | 1 | Mild | None | None | Complete recovery/return to baseline condition |
| 16 | 4 | Deterioration of suicidal ideation | No | No | Yes | N/A | 2 | Mild | None | Psychotherapy | Complete recovery/return to baseline condition |
| 17 | 5 | Deterioration of suicidal ideation | No | No | Yes | N/A | 1 | Mild | None | Psychotherapy | Complete recovery/return to baseline condition |
| 18 | 6 | Deterioration of suicidal ideation | No | No | Yes | N/A | 9 | Mild | None | None | Complete recovery/return to baseline condition |
| 19 | 1 | Headache | No | No | Yes | No | 1 | Mild | None | Non-prescription drug | Complete recovery/return to baseline condition |
| 20 | 1 | Headache | No | No | Yes | No | 1 | Moderate | None | Non-prescription drug | Complete recovery/return to baseline condition |
| 21 | 2 | Diarrhea | No | No | Yes | No | 2 | Moderate | None | None | Complete recovery/return to baseline condition |
| 22 | 3 | Anxiety | No | No | Yes | No | ≥94 days, continuing at least until the study termination | Moderate | None | Prescription drug (diazepam and zopiclone) | Persisting, diminishing |
| 23 | 4 | Deterioration of suicidal ideation | No | No | Yes | No | ≥71 days, continuing at least until the study termination | Moderate | None | Psychotherapy (extra visit on site and on phone) | Persisting, diminishing |
| 24 | 5 | Headache | No | No | Yes | No | 1 | Moderate | None | Non-prescription drug | Complete recovery/return to baseline condition |
| 25 | 6 | Jaw muscle tightness | No | No | Yes | No | 1 | Mild | None | None | Complete recovery/return to baseline condition |
| 26 | 1 | Headache | No | No | Yes | No | 1 | Mild | None | Non-prescription drug | Complete recovery/return to baseline condition |
| 27 | 2 | Loss of appetite | No | No | Yes | No | 1 | Mild | None | None | Complete recovery/return to baseline condition |
| 28 | 3 | Dry mouth | No | No | Yes | No | 1 | Mild | None | None | Complete recovery/return to baseline condition |
| 29 | 1 | Nausea | No | No | Yes | No | 1 | Moderate | None | None | Complete recovery/return to baseline condition |
| 30 | 2 | Hyperhidrosis | No | No | Yes | No | 1 | Mild | None | None | Complete recovery/return to baseline condition |
| 31 | 3 | Nausea | No | No | Yes | No | 1 | Mild | None | Psychotherapy | Complete recovery/return to baseline condition |
| 32 | 4 | Jaw muscle tightness | No | No | Yes | Yes | 2 | Mild | None | Psychotherapy | Complete recovery/return to baseline condition |
| 33 | 5 | Nystagmus | No | No | Yes | No | 1 | Mild | None | None | Complete recovery/return to baseline condition |
| 34 | 6 | Deterioration of suicidal ideation | No | No | Yes | N/A | 7 | Mild | None | Psychotherapy | Complete recovery/return to baseline condition |
| 35 | 7 | Deterioration of suicidal ideation | No | No | Yes | N/A | 1 | Mild | None | Psychotherapy | Complete recovery/return to baseline condition |
| 36 | 8 | Deterioration of suicidal ideation | No | No | Yes | N/A | 7 | Mild | None | Psychotherapy | Complete recovery/return to baseline condition |
| 37 | 9 | Deterioration of suicidal ideation | No | No | Yes | N/A | 22 | Mild | None | Psychotherapy | Complete recovery/return to baseline condition |
| 38 | 1 | Headache | No | No | Yes | No | 1 | Mild | None | None | Complete recovery/return to baseline condition |
| 39 | 1 | Headache | No | No | Yes | No | 1 | Mild | None | Non-prescription drug | Complete recovery/return to baseline condition |
| 40 | 2 | Hypoesthesia | No | No | Yes | No | 1 | Mild | None | None | Complete recovery/return to baseline condition |
| 41 | 3 | Dry mouth | No | No | Yes | No | 1 | Mild | None | None | Complete recovery/return to baseline condition |
| 42 | 4 | Blurred vision | No | No | Yes | No | 1 | Mild | None | None | Complete recovery/return to baseline condition |
| 43 | 5 | Tremor | No | No | Yes | No | 2 | Mild | None | Psychotherapy | Persisting, diminishing |
| 44 | 6 | Anxiety | No | No | Yes | Yes | 2 | Moderate | None | Psychotherapy and prescription drug (diazepam) | Complete recovery/return to baseline condition |
| 45 | 7 | Jaw muscle tightness | No | No | Yes | No | 1 | Mild | None | None | Complete recovery/return to baseline condition |
| 46 | 1 | Deterioration of suicidal ideation | No | No | Yes | N/A | 7 | Mild | None | None | Complete recovery/return to baseline condition |
