# Supplementary material for: MDMA-assisted therapy as a treatment for major depressive disorder: proof of principle study
Source: Br J Psychiatry. 2025 Jul 11;227(5):783–9. doi: 10.1192/bjp.2025.10320 (PMC12550655; doi:10.1192/bjp.2025.10320)
Supplement: Kvam et al. supplementary material 11 — Kvam et al. supplementary material [file S0007125025103206sup011.docx]

# Methods

## Participants

*Exclusion criteria* were current or previously diagnosed psychotic disorder, significant history of mania, personality disorder, eating disorder with active purging, alcohol or substance use disorders, serious suicide risk, pregnancy or breastfeeding, electroconvulsive therapy or ketamine within 12 weeks of enrollment, or history of any medical condition that could make receiving a sympathomimetic drug harmful because of increases in blood pressure and heart rate, including uncontrolled hypertension, history of arrhythmia, prolongation of QT/QTc interval at screening, history of myocardial infarction, cerebrovascular incident, or aneurysm [1]. The participants would also need to comply with specific lifestyle modifications, such as the tapering and discontinuation of certain medications prior to the dosing sessions, and to remain overnight at the study site after each dosing session.

## Study Drug

The study drug was manufactured in accordance with Current Good Manufacturing Practice (CGMP) standards by Onyx Scientific. The investigational medicinal product  (IMP) was obtained from Lykos. Sharp Clinical Services was the manufacturer responsible for the batch release from the US to the EU. The IMP was shipped to Norway via an EU depot in Slovakia. SanaClis has the license to manufacture, import, export, and store the IMP.

## Approvals

The study was approved by the Regional Committees for Medical and Health Research Ethics (application number 145565) and the Norwegian Medicines Agency (reference number 21/18379-18). Authorization to prescribe the study medication was obtained from the Norwegian Directorate of Health (case number Case 22/8615).

## Procedures

### Pre-study

To assess eligibility, the participants were first phone screened. If deemed initially eligible, the participants visited the study site for signing the Informed Consent Form (ICF) after the procedures had been full explained, and an extended screening. The screening measures included the Beck’s Depression Inventory II (BDI-II), the Colombia-Suicide Severity Rating Scale (C-SSRS; both lifetime and for the past six months at the screening visit; and the since last visit version at all subsequent visits), the Mini International Neuropsychiatric Interview for DSM-5 (MINI), the Structured Clinical Interview for DSM-5 Screening Personality Questionnaire and Structured Clinical Interview for DSM-5 Personality Disorders (SCID-5-SPQ and -PD), Alcohol Use Disorders Identification Test (AUDIT) and Drug Use Disorders Identification Test (DUDIT). Additionally, we mapped previous psychotherapy and MDMA use, psychiatric and medical history, as well as pre-study medications and adherence. The study team obtained medical records from general practitioners and specialists and hospitals where available, conducted a physical examination, clinical laboratory testing (including pregnancy and drug tests), electrocardiogram, and 1-min rhythm strip. The physical exams and assessments for eligibility were conducted by two physicians trained as general practitioners, one of whom was also a specialist in internal medicine. Psychotropic medications, if any, were tapered post-enrollment and discontinued at least five half-lives plus at least 1 week for stabilization before the baseline assessments in accordance with the protocol. Clinical data were collected using the electronic case report form provided by Ledidi.

### Retention

Screen failures were considered potentially eligible during phone screen, but were not enrolled in the study as they did not meet the criteria for enrollment post-ICF. Pre-dosing early terminations were initially enrolled, but did not qualify during the enrollment confirmation before the first MDMA session. Post-dosing early terminations were defined as those who wanted to stop treatment, but who attend study visits and participate in outcome assessments. Dropouts were participants who withdrew their consent and chose not to participate in any additional study visits or measurements. Lost to follow-up refers to participants who failed to attend study visits and could not be reached by the site staff.

### Preparatory period and enrollment confirmation

The main purpose of the three 90-minutes preparatory sessions was to establish a therapeutic alliance, and help the participants feel a sense of safety and intrapersonal and interpersonal trust. As a preparation for the MDMA dosing sessions, the participants were encouraged to face difficult memories and emotions rather than to avoid or suppress them, and to stay present with whatever that emerged in the MDMA sessions in order to explore, process and analyze the material.

### MDMA-assisted therapy

MDMA-AT aims to create an effective working alliance with the participant, working in an “inner-directed way”, encouraging exploration and self-inquiry rather than providing a direction or explanation [2]. The therapist dyad encourages the participant to focus internally, and explore whatever thoughts, emotions and memories that surface. In case the participant feels stuck or unable to continue the exploration, the therapists provide validating or reassuring statements, invite the participant to use the breath to explore and express the emerging material, or suggest the use of nurturing touch. All instances of nurturing touch, like the therapists placing a hand on the participant’s shoulder or offering a hand, were discussed with participants in advance, and only applied with their consent prior to MDMA dosing. The purpose of the integration sessions was to continue the processing of the unfolding experience post-MDMA, and to apply any new insights, skillsets or attitudes into daily life.

Psychotherapists

Three of the therapists were trained prior to a Lykos-sponsored trial investigating MDMA-AT for PTSD [3]. This 100 hour (+ supervision) training consisted of reading the Treatment Manual [2], online training modules, a training course that included watching and discussing videos of MDMD-AT from previous clinical trials, experiential learning, role playing, and supervision.

## Adverse events

The study protocol had a special attention to a subset of adverse events (AEs), namely adverse events of special interest (AESIs). The following were considered as AESIs and marked as such in the eCRF: 1) symptoms that could be indicative of QT interval prolongation or cardiac arrhythmia. 2) Increased suicide risk including suicides, suicide attempts, self-injurious behavior associated with suicidal ideation, suicidal ideation scores of 4 or 5 on the C-SSRS, and suicidal ideation judged to be serious or severe in the opinion of the study team. 3) Information indicative of abuse potential, including Behavioral addiction, Drug abuser, Substance abuser, Dependence, Intentional product misuse, Overdose (accidental, intentional, or prescribed), or Drug diversion of MDMA or “Ecstasy”.

# Results

## Feasibility

We found the phone screens to be useful, as they allowed us to conduct a broad initial screening before participants underwent the more resource-intensive on-site screening.

Pre-dosing early terminations were due to: one participant’s baseline MADRS score being too low, another was excluded based on difficulty in forming a working alliance, and a third was excluded for lack of appropriate social support.

# References

1. Kvam, T.-M., et al., *Study protocol for “MDMA-assisted therapy as a treatment for major depressive disorder: A proof of principle study”.* Frontiers in Psychiatry, 2022. **13**.

2. Mithoefer, M.C., *A Manual for MDMA-Assisted Psychotherapy in the Treatment of Posttraumatic Stress Disorder;*. 2016.

3. ClinicalTrials.gov. U.S. National Library of Medicine.
